# HDAC5-mediated Smad7 silencing through MEF2A is critical for fibroblast activation and hypertrophic scar formation

**DOI:** 10.7150/ijbs.76140

**Published:** 2022-09-11

**Authors:** Ya Gao, Yangdan Liu, Danning Zheng, Chiakang Ho, Dongsheng Wen, Jiaming Sun, Lu Huang, Yuxin Liu, Qingfeng Li, Yifan Zhang

**Affiliations:** Department of Plastic & Reconstructive Surgery, Shanghai Ninth People's Hospital, School of Medicine, Shanghai Jiao Tong University, Shanghai, China.

**Keywords:** Hypertrophic scar, HDAC5, TGF-β, Smad7, MEF2A

## Abstract

Transforming growth factor-β (TGF-β) signaling plays a key role in excessive fibrosis. As a class IIa family histone deacetylase (HDAC), HDAC5 shows a close relationship with TGF-β signaling and fibrosis. However, the effect and regulatory mechanism of HDAC5 in hypertrophic scar (HS) formation remain elusive. We show that HDAC5 was overexpressed in HS tissues and depletion of HDAC5 attenuated HS formation *in vivo* and inhibited fibroblast activation *in vitro*. HDAC5 knockdown (KD) significantly downregulated TGF-β1 induced Smad2/3 phosphorylation and increased Smad7 expression. Meanwhile, Smad7 KD rescued the Smad2/3 phosphorylation downregulation and scar hyperplasia inhibition mediated by HDAC5 deficiency. Luciferase reporter assays and ChIP-qPCR assays revealed that HDAC5 interacts with myocyte enhancer factor 2A (MEF2A) suppressing MEF2A binding to the Smad7 promoter region, which results in Smad7 promoter activity repression. HDAC4/5 inhibitor, LMK235, significantly alleviated hypertrophic scar formation. Our study provides clues for the development of HDAC5 targeting strategies for the therapy or prophylaxis of fibrotic diseases.

## Introduction

Hypertrophic scar (HS) is a fibro-proliferative disorder with the accumulation of abnormal extracellular matrix (ECM) that forms within the original wounded area that develops from burns, surgeries or traumatic injuries 1, 2. HS not only leads to compromised cosmetic outcomes but also induces functional impairment that seriously affects the social function and quality of life of patients 3. Considering its role in physical, mental and social health, HS has been identified as one of the significant unmet functionally and psychosocially challenges to global health 4. However, the detailed mechanism is not fully understood, and there has been no breakthrough in anti-scar therapy. Studies investigating the molecular mechanism of hypertrophic scar formation and targeted intervention are thus worthwhile and promising.

Transforming growth factor-β (TGF-β) signaling has been well recognized as a key regulator of HS formation 5. It exerts pleiotropic effects on different phases of wound repair by regulating fibroblast activation, ECM production, cell proliferation and migration, epithelial-mesenchymal transition (EMT), and the immune response 6, 7. Thus, multiple attempts have been made to regulate TGF-β signaling for the treatment of aberrant skin wound repair and HS formation 8, 9. A previous study demonstrated that the early application of neutralizing antibodies to TGF-β1/2 can reduce the collagen and fibronectin contents, thus attenuate scar formation 10. However, subsequent studies found that topical inhibition of TGF-β signaling may be related to non-healing wounds 11. Additionally, as a central signaling cascade involved in multiple cellular processes, systemic inhibition of the TGF-β pathway frequently leads to a mixed inflammatory cell response and tissue necrosis, resulting in organ failure and death 9.

Recently, histone deacetylases (HDACs) have been reported to play a prime role in regulating intermediates of the TGF-β pathway. One study showed that HDAC1 can form an SP1-SIN3A-CoREST-LSD1-HDAC1 complex that binds to the promoter region of TGF-β pathway target genes and inhibits TGF-β pathway activation^12^, suggesting that epigenetics plays an important role in fibrosis 13. Barter MJ *et al.* demonstrated that depletion of HDAC3 markedly blocked the activation of ERK and PI3K by TGF-β1 in osteoarthritis cells 14. In addition, HDAC7 has been found to be involved in the repression of key genes required for TGF-β-mediated fibroblast activation 15. On the other hand, HDACs have been proven to be closely related to fibrosis in various tissues and organs. A study suggested that cardiac fibroblast activation is controlled by crosstalk between HDACs and BRD4, with HDAC inhibition blocking the recruitment of BRD4 to profibrotic gene regulatory elements 16. Researchers have also shown that patients with chronic obstructive pulmonary disease have a progressive reduction in total HDAC activity that reflects the severity of the disease 17. Several clinical studies using HDAC inhibitors (HDACi) for fibrosis are also underway. A phase-II clinical trial showed that a pan-HDACi, Mocetinostat (MGCD0103, MOCE), alleviated myocardial fibrosis 18. Researchers conducted a single-institution, phase-II, investigator-initiated trial of ruxolitinib plus pracinostat (pan-HDACi) in 25 patients with myelofibrosis, which demonstrated that sixteen (80%) patients had objective responses 19. Thus, as an important regulator of TGF-β signaling, HDACs are promising targets for fibrosis treatment.

HDAC5, a class IIa family HDAC, often confers responsiveness to external signals and allows inhibitory binding to myocyte enhancer factor 2 (MEF2) family transcription factors. A recent study showed that HDAC5-KD significantly downregulated TGF-β1 expression and that the HDAC5-TGF-β1 axis was involved in the epithelial-mesenchymal transition (EMT) 20. Using MC1568 (class IIa-specific HDACi) or siRNAs targeting HDAC5 led to activation of Smad-dependent downstream gene expression 21. In addition, bisphenol A downregulated H3K9ac and the expression levels of TGF-β via the ERβ/HDAC5 signaling pathway. Hence, HDAC5 is closely related to the TGF-β pathway. Another study focusing on systemic sclerosis (SSc) showed that HDAC5 can regulate fibrosis-related genes 22. Researchers also found that knocking down HDAC5 and HDAC6 via a gene-editing strategy dramatically blocked Ang II-induced hypertrophic responses in cardiac hypertrophy and fibrosis 23. However, how HDAC5 regulates the TGF-β pathway and how this is related to skin fibrosis deserve further investigation.

In the present study, we found that HDAC5 was increased both in human and mouse HS tissue. HDAC5 knockout (KO) mice exhibited milder HS formation. Furthermore, we revealed that by interacting with the transcription factor MEF2, HDAC5 inhibited Smad7 expression, which in turn mediated TGF-β-induced fibroblast activation. Inhibition of HDAC5 by shRNA or an inhibitor increased MEF2-mediated Smad7 expression, suppressed TGF-β-induced fibroblast activation and attenuated HS formation. By manipulating the target HDAC5, we hope to provide a new treatment strategy for HS and other TGF-β1-modulated fibroproliferative diseases.

## Materials and Methods

### Ethics approval and human tissue samples

Twenty normal skin tissues and hypertrophic scar tissues were acquired in plastic surgery from Shanghai Ninth People's Hospital with ethics approval from local Human Research Ethics Committee of Shanghai Jiao Tong University School of Medicine in accordance with the Declaration of Helsinki principles. Written informed consent was obtained from patients. Primary human hypertrophic scar fibroblasts (HSFs) were isolated using the hypertrophic scar tissues. Volunteer information was listed in Supplementary Table S1.

### Animal ethics

Animal welfare were strictly adhered to the principles of “Guide for the care and use of laboratory animals” (National Research Council. National Academies Press; 27 December 2010). Mice were housed under standard conditions and all procedures were performed in accordance with Guide for the Care and Use of Laboratory Animals approved by the Committee on the Ethics of Animal Experiments of Shanghai Jiao Tong University School of Medicine. And at the end of *in vivo* experiment, euthanasia was conducted according to “CCAC guidelines on: euthanasia of animals used in science. Canadian Council on Animal Care”.

### Animals

Female C57BL/6 mice which were eight weeks old were purchased from Shanghai Slac Laboratory Animal (Slac, Shanghai, China). HDAC5 KO mice were generated by CRISPR/Cas9 system conducted by Biocytogen Pharmaceuticals (Beijing) Co., Ltd using C57BL/6 mice. Single guide RNAs (sgRNAs) were designed targeting the exon 6 to 29 of HDAC5 and were co-injected with Cas9 into the zygotes. The pups obtained were genotyped by PCR. After genotyping, the F0 mice went through serial mating to generate homozygous mutant offspring.

### Hypertrophic scar model

The load-induced hypertrophic scar model was proceeded based on a model which was built by Geoffrey C Gurtner etc. 24. In brief, on day 1, a 2 cm liner incision was made on the dorsal midline of the mice and reapproximated with 6-0 nylon sutures. On post incision day 4, sutures were removed from the scars and a biomechanical loading device was carefully secured with 6-0 nylon sutures. Mechanical load on the scars was created by carefully distracting the expansion screws of the devices by 2 mm on day 4 and by 4 mm every other day thereafter until 2 weeks to maintain the pressure.

Both wide-type (WT) mice and HDAC5 KO mice were loaded according to the method described above. Half the mice in each group were sacrificed to harvest the scars on day 14 and the other half were observed on day 21. In AAV-virus injection experiments, HDAC5 KO mice were randomly divided into 2 groups, including the loaded+AAV5-shCtrl group and the loaded+AAV5-shSmad7 group. The time schedule was followed the procedures of the previous experiment.

In inhibitors injection animal experiments, LMK235 (Selleck, S7569), a HDAC4/5 inhibitor, was diluted using 5%DMSO+30%PEG300+5%TW80+ddH_2_O and the injection dose is 1 mg/kg. HDAC4 inhibitor-Tasquinimod (Selleck, S7617) was diluted using 5%DMSO+30%PEG300+ddH_2_O and the injection dose is 1 mg/kg. A total of 54 mice were equally randomized into three groups: the vehicle-treated group, the LMK235-treated group and the Tasquinimod-treated group. Inhibitors were injected into the subcutaneous of mice dorsal skin 5 days before mechanical loading and continuously injected during the loading period. We administered daily subcutaneous injections (100 µl) of the solvent or LMK235 or Tasquinimod to the wound area of the mice. The injection sites were outside the wound area and were changed every day, but the vehicle or inhibitors was diffused within the wound area.

### Cells

Human HS-derived fibroblasts (HSFs) isolation steps are described in our previous study 25. After excision, use sterile 1×PBS to wash the scar sample three times, then put it into 0.25% trypsin solution overnight at 4 °C. Next day remove the epidermis, and cut scar to small pieces. Then use 0.25% Collagenase IV solution digest at 37 °C for 4 h. Passing through 200-mesh sieve. Centrifuge 5 min, 1000 r/min. Mouse embryonic fibroblasts (MEFs) preparation and culture were as follows. Briefly, embryos were harvested on embryonic day 13.5-14.5, after the heads and most of the internal organs were removed, the remaining tissues were put into 0.25% trypsin-EDTA overnight at 4 °C and were digested for 30 min at 37 °C on the next day. Cells were cultured in Dulbecco's Modified Eagle Medium (DMEM) (Gibco, USA) supplemented with 10% fetal bovine serum (FBS) (Gibco, USA) and 1% penicillin/streptomycin antibiotics (Gibco, USA). In some groups cells were treated with TGF-β1 (R&D system, 7754-BH) at 5 ng/mL.

### RNA purification and quantitative real-time PCR (RT-qPCR)

Total RNA was extracted using TRIzol reagent (Invitrogen). RT-qPCR was performed with an ABI 7900HT system using SYBR Premix (Takara, Dalian, China) according to the manufacturer's instructions. mRNA quantification was performed using glyceraldehyde 3-phosphate dehydrogenase (GAPDH) for normalization. The primers used in this study were listed in Supplementary Table S2.

### Western blot assay

Tissues and cultured cells were lysed for 30 min with RIPA lysis buffer supplemented with protease inhibitor (Roche, Mannheim, Germany). To analyze inducible protein expression, 20 μg protein was resolved by 10% or 12% sodium dodecyl sulfate-polyacrylamide gel electrophoresis (SDS-PAGE) and electroblotted in polyvinylidene difluoride (PVDF) membranes (Millipore, Bedford, MA, USA). The membranes were blocked with 5% nonfat milk at room temperature for 1 hour. The separated proteins were then incubated with primary antibodies: anti-GAPDH (CST, #5174, 1:1000), anti-HDAC5 (Invitrogen, PA1-41117, 1:1000), anti-Col1a1 (CST, #72026, 1:1000), anti-Col3a1 (NOVUS, NBP1-05119, 1:500), anti-Smad2 (Abcam, ab33875, 1:1000), anti-p-Smad2 (Abcam, ab280888, 1:1000), anti-Smad3 (Abcam, ab208182, 1:1000), anti-p-Smad3 (Abcam, ab52903, 1:2000), anti-Smad4 (Abcam, ab40759, 1:5000), anti-Smad6 (Abcam, ab273106, 1:1000), anti-Smad7 (Santa Cruz, sc-365846, 1:1000), anti-TGFβRI (Abcam, ab235578, 1:1000), anti-TGFβRII (Abcam, ab259360, 1:1000), anti-Gremlin-1 (Santa Cruz, sc-515877, 1:500), anti-MEF2A (Santa Cruz, sc-17785, 1:1000), anti-Ac-lysine (Santa Cruz, sc-32268, 1:500) antibodies at 4 °C overnight. The membranes were incubated with peroxidase-conjugated secondary antibody at room temperature on the next day. Quantitative analysis was performed on the immunoreactive bands with Image J software.

### Histology and immunohistochemistry

Tissues that were paraformaldehyde-fixed overnight and then paraffin-embedded and sliced. Sections were stained with hematoxylin and eosin (H&E) or Picrosirius red. For immunohistochemistry staining, sections were incubated with primary antibody against HDAC5 (Abcam, ab55403, 1:200) or α-SMA (Santa Cruz, sc-53142, 1:200) overnight at 4 °C and on the next day, sections were incubated with HRP-conjugated secondary antibody and counterstained with hematoxylin and developed with diaminobenzidine. Image-Pro Plus 6.0 software was used for quantitative analysis.

### Immunofluorescence

For immunofluorescent staining, tissue sections or cells were incubated with primary antibody against α-SMA (Santa Cruz, sc-53142, 1:200) or HDAC5 (Abcam, ab55403, 1:200) overnight at 4 °C, followed by the appropriate secondary antibody. Nuclei were stained with 4′,6-diamidino-2-phenylindole. Fluorescence was analyzed using a Zeiss 710 laser-scanning microscope (Zeiss, Thornwood, NY, USA). Immunofluorescence co-localization analysis was performed using Image J software.

### shRNA and plasmid transfection

For HDAC5 silencing, HSFs were transfected with HDAC5 shRNA (Santa Cruz Biotechnology, sc-35542-SH). For Smad7 silencing, HDAC5 KD HSFs were transfected with Smad7 shRNA (Santa Cruz Biotechnology, sc-36508-SH), HDAC5 KO MEFs were transfected with Smad7 shRNA (Santa Cruz Biotechnology, sc-36509-SH). All transfections were using Lipofectamine 3000 reagent (ThermoFisher Scientific, #L3000150) according to the manufacturer's protocol. Nontargeting shRNA plasmid (Santa Cruz Biotechnology, sc-108060) was used as a negative control.

For Co-IP assay, cDNAs of HDAC5, Smad7, MEF2A and p65 were subcloned into pDONR201 (Invitrogen) following the manufacturer's protocol as entry clones. The insert of the resulting pDONR clone was verified by sequencing. And subsequently transferred to gateway-compatible destination vectors as previously described 26.

### 5-Ethynyl-2′-deoxyuridine (EdU) proliferation assay

Cells seeded in 24-well plates were incubated under standard conditions and were divided into different groups. 24 h after incubation, cell proliferation was detected using the incorporation of 5-ethynyl-2-deoxyuridine (EdU) with the EdU Cell Proliferation Assay Kit (Invitrogen, Click-iT^®^ EdU Imaging Kits C10337). Steps were conducted according to the manufacturer's protocol. Briefly, the cells were incubated with 50 μM EdU for 2 h before fixation, permeabilization and EdU staining. Then cell nuclei were stained with DAPI (Sigma-Aldrich, D9542) at a concentration of 1 μg/mL for 8 min. The proportion of cells that incorporated EdU was determined by Zeiss 710 laser-scanning microscope (Zeiss, Thornwood, NY, USA).

### *In vitro* wound-healing assay

Cells were seeded in 6-well plates to near confluence. Wounds were generated using a sterilized micropipette tip. Wound healing tests were performed in complete medium and photographed at 0 and 12 h. To quantify cell migration, the wound width from 10 randomly selected areas were measured at each time point, and the migration distance was the difference in width between 0 and 12 h. The migration distance of each sample was first normalized to the initial wound width and then compared with each other.

### Collagen gel contraction assay

Cells were seeded in 24-well plates in 500 μL of collagen suspension (IBFB, Leipzig, Germany). After collagen gel polymerization, the gels were released immediately from plates by tilting plates slightly. The area of each collagen gel was measured at day 3. Statistical analysis was performed using Image J software.

### AAV (adeno-associated virus) vector administration

We utilized the AAV Helper-Free System (AAV Helper-Free System, Stratagene) for viral production using a triple-transfection, helper-free method and purified it as described in a previous study. The interference sequences were as follows: Smad7 shRNA: 5ʹ-CACCGCTTTCAGATTCCCAACTTCTCGAAAGAAGTTGGGAATCTGAAAGC-3ʹ and control shRNA, 5′-TTCTCCGAACGTGTCACGT-3′. Mice were anaesthetized with an isoflurane/air mix (3% for initial induction and 1.5-2% for maintenance). Three hundred nanoliters of either AAV5-shSmad7 or AAV5-shCtrl was injected into the subcutaneous of mice dorsal skin 5 days before mechanical loading and continuously injected during the loading period according to different groups. The injections were performed using a 34-gauge needle (World Precision Instruments) attached to a 10-μL NanoFil microsyringe (Nanofil, World Precision Instruments).

### Co-immunoprecipitation (Co-IP) assay

According to the needs of different Co-IP experiments, related proteins were overexpressed through gateway system. Briefly, cell lysates were collected using ice-cold IP lysis buffer. The lysates were transferred to a micro centrifuge tube and centrifuged at 13,000×g for 10 min. The supernatant was transferred to a new tube for protein concentration determination and further analysis. The Co-IP analyses were performed using a Co-Immunoprecipitation Kit (ThermoFisher Scientific, #26149) according to the manufacturer's protocol. The experimental steps including: pre-clear lysate using the control agarose resin, Co-IP, elution of Co-IP, resin regeneration and preparation for SDS-PAGE analysis were carried out in turn. Antibodies used therein include: anti-HDAC5 antibody (Invitrogen, PA1-41117, 1:500), anti-Smad7 antibody (Santa Cruz, sc-365846, 1:200), anti-MEF2A antibody (Santa Cruz, sc-17785, 1:200) and anti-NF-κB p65 antibody (Santa Cruz, sc-8008, 1:200). Normal rabbit IgG without antigenicity provided with the kit was used as a negative control. SDS-PAGE and immunoblotting were performed as described above.

### Chromatin immunoprecipitation (ChIP) assay

ChIP assay was performed using Millipore Chip Kit (#17-10085) according to the manufacturer's protocol. Briefly, cells cultured under the previously indicated conditions were fixed in 1% formaldehyde/PBS for 10 min at room temperature. After two washes with PBS, cells were resuspended in 0.5 mL of lysis buffer containing a protease inhibitor cocktail before sonication. DNA fragments from the soluble chromatin preparations were 400-800 bp in length. Immunoprecipitation was carried out overnight with purified anti-MEF2A antibody (Santa Cruz, sc-17785, 1:200) or anti-NF-κB p65 antibody (Santa Cruz, sc-8008, 1:200) or normal rabbit IgG as a negative control according to a previous study 27. Protein A/G agarose was used to pull down the antigen-antibody compounds and then washed four times with washing buffers. The DNA-protein crosslinks were reversed with 5 M NaCl at 65 °C for 6 h, and DNA from each sample was purified. PCR was performed using 2 μL DNA samples with the following primers: Smad7: forward 5′-GAATCTTACGGGAAGATCAAC-3′, reverse 5′-CGCAGAGTCGGCTAAGGT-3′.

### Luciferase reporter assay

Oligonucleotides used in the construction of the Smad7 luciferase reporter plasmid was designed. The luciferase reporter containing the Smad7 promoter (-1400; +112) were conducted as previously reported 28, forward primers: 5'-AATTGAGCTCGGGAGGGAAGGGGGCGGG-3'. All PCR products were subcloned into pGL3-Basic (Promega). The reporter construct was further mutated at the site1/2 with a QuikChange™ ⅡSite-Directed Mutagenesis kit (Stratagene, La Jolla, CA, USA) according to the manufacturer's instruction. pFA2-MEF2A expression plasmid was generated. Briefly, for the reporter assay, cells were plated at density of 8 × 10^4^ cells per well in 6-well plates 1 day before transfection. The pGL3 Smad7 (WT/mutsite1/mutsite2)-Luc and the pFA2-MEF2A/pFA2-vector (control) were transfected using FuGENE 6 (Roche) according to the manufacturer's instructions on individual experiments (see figure legends for details). For the gene overexpression constructs for HDAC5 refer to the shRNA and plasmid transfection section. Then the cells were lysed and luciferase reporter activity was measured using the Luciferase Reporter system (Promega) with Firefly luciferase values normalized to Renilla luciferase values.

### Statistical analysis

Statistical software package SPSS 20.0 (SPSS, Chicago, IL, USA) was used for statistical analysis. An independent-samples *t* test was used to evaluate differences between two groups. One-way analysis of variance (ANOVA) followed by a post hoc test (Tukey test) was used to compare two groups in multiple comparisons. Normally distributed data are expressed as the mean ± standard deviation (SD), and asymmetrically distributed data are expressed as median (range). We set *P* < 0.05 as the threshold of statistical significance in all statistical analyses. All experiments were repeated at least three times.

## Results

### HDAC5 is overexpressed in HS tissues

**Figure 1:**
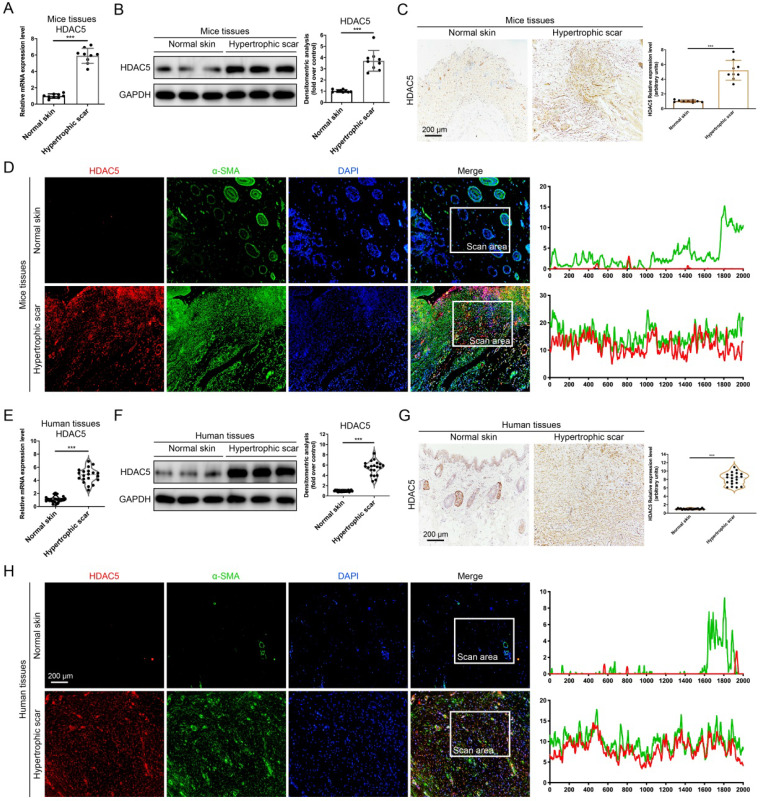
** HDAC5 is overexpressed in mice and human HS. (A, B)** The mRNA and protein levels of HDAC5 in normal mouse skin and HS tissues. **(C)** Images and quantitative analysis of immunohistochemical staining of HDAC5 in normal skin and HS tissues of mice. (Scale bar = 200 µm). **(D)** Immunofluorescence colocalization assay of HDAC5 and α-SMA in normal skin and HS tissues of mice. HDAC5 is labeled in red, and α-SMA is labeled in green. (Scale bar = 200 µm). **(E, F)** The mRNA and protein levels of HDAC5 in normal human skin and HS tissues. **(G)** Images and quantitative analysis of immunohistochemical staining of HDAC5 in normal skin and HS tissues of humans. (Scale bar = 200 µm). **(H)** Immunofluorescence colocalization assay of HDAC5 and α-SMA in normal skin and HS tissues of humans. HDAC5 is labeled in red, and α-SMA is labeled in green (Scale bar = 200 µm). Data are presented as the mean ± SD (n = 9 biologically independent animals and n = 20 biologically independent humans). ***P < 0.001.

Several histone deacetylases have been reported interacted with TGF-β1 12, 14, 15 and associated with tissue fibrosis 15. Therefore, we detected the mRNA expression of HADC family members, including HDAC 1-11 and SIRT 1-7, in a mouse HS model. Our data demonstrated that among these HADC family members, HDAC5 is highly expressed in HS tissues compared with other HADCs (Supplementary Fig. S1 and Fig. 1A). Meanwhile, Western blot and immunohistochemistry staining results further confirmed that the protein levels of HDAC5 were significantly increased in HS tissues compared with normal skin tissues (Fig. 1B, C). In addition, immunofluorescence staining revealed the colocalization of HDAC5 and α-SMA, a biomarker of myofibroblasts, which demonstrated that HDAC5 was highly expressed in activated fibroblasts in mouse HS tissues (Fig. 1D). More importantly, the expression level of HDAC5 was also determined in human tissues. In line with our mouse results, HDAC5 expression levels were significantly higher in human HS tissues than in normal skin (Fig. 1E-G), and immunofluorescence staining revealed the co-localization of HDAC5 with α-SMA (Fig. 1H). These results suggested that HDAC5 might be associated with HS formation.

### HDAC5 knockout attenuates HS formation *in vivo*

**Figure 2:**
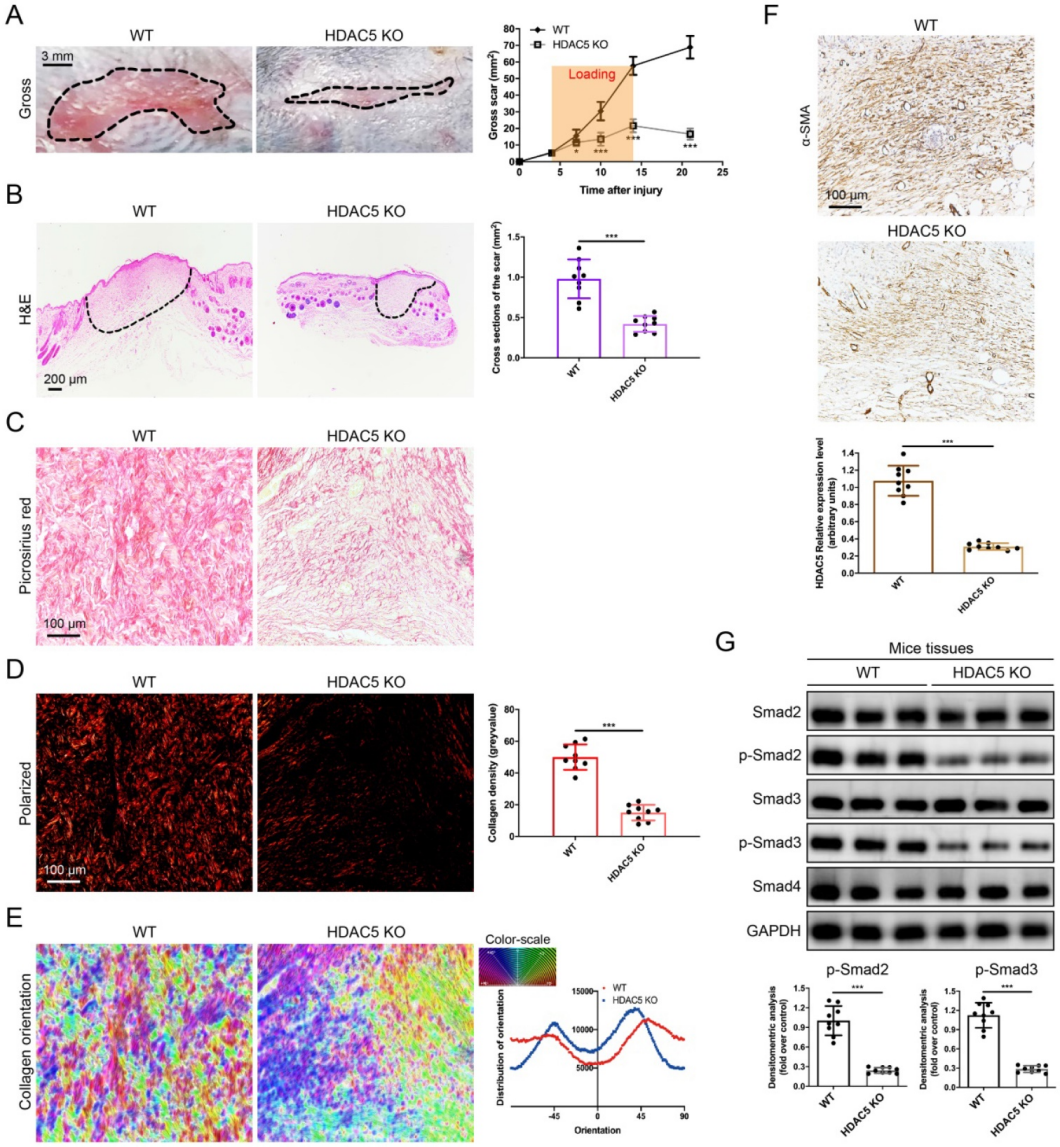
** HDAC5 knockout attenuates hypertrophic scar formation *in vivo*. (A)** Images of scars 14 days postincision and gross area quantification at all examined time points. (Scale bar = 3 mm). **(B)** Images of H&E-stained sections and cross-section size quantification. The dashed lines outline the scar. (Scale bar = 200 µm). **(C, D)** Images of picrosirius red-stained sections under ordinary light and polarized light and collagen density quantification. (Scale bar = 100 µm). **(E)** The orientation of collagen fibers was quantified from picrosirius red using Orientation J software. The color representation reflects the different orientations. **(F)** Images and quantitative analysis of immunohistochemical staining of α-SMA in HS tissues. (Scale bar = 100 µm). **(G)** Western blot assay of phosphorylated and total Smad2 and Smad3 and total Smad4 in HS tissues. Data are presented as the mean ± SD (n = 9 biologically independent animals). *P < 0.05, ***P < 0.001.

To further study the roles of HDAC5 in HS formation, we constructed HDAC5 KO mice with the CRISPER/Cas9 system (Supplementary Fig. S2). Consistent with our assumption, HDAC5 KO mice showed attenuated scar formation with a significantly reduced gross scar area at each examined time point (Fig. 2A). Further histological analysis demonstrated that the cross-sectional size of the scar in HDAC5 KO mice was markedly decreased at Day 14 (Fig. 2B). Moreover, to evaluate collagen deposition in HS tissues, picrosirius red staining was conducted. Quantitative analysis showed a dramatically reductive collagen density (Fig. 2C, D) and a substantially decreased disorder in the collagen fibril orientation (Fig. 2E) in HS tissues of HDAC5 KO mice vs. WT mice. Our results also demonstrated that the expression levels of α-SMA, the marker of activated fibroblasts, were significantly reduced in HS tissues of HDAC5 KO mice vs. WT mice (Fig. 2F). Besides, phosphorylation of Smad2 and Smad3 was significantly blocked in HS tissues of HDAC5 KO mice without having an obvious impact on total Smad2/3 and Smad4 expression (Fig. 2G). These data indicated that HDAC5 KO significantly inhibited scar hypertrophy.

### HDAC5 knockdown inhibits TGF-β1-induced fibroblast activation *in vitro*

**Figure 3:**
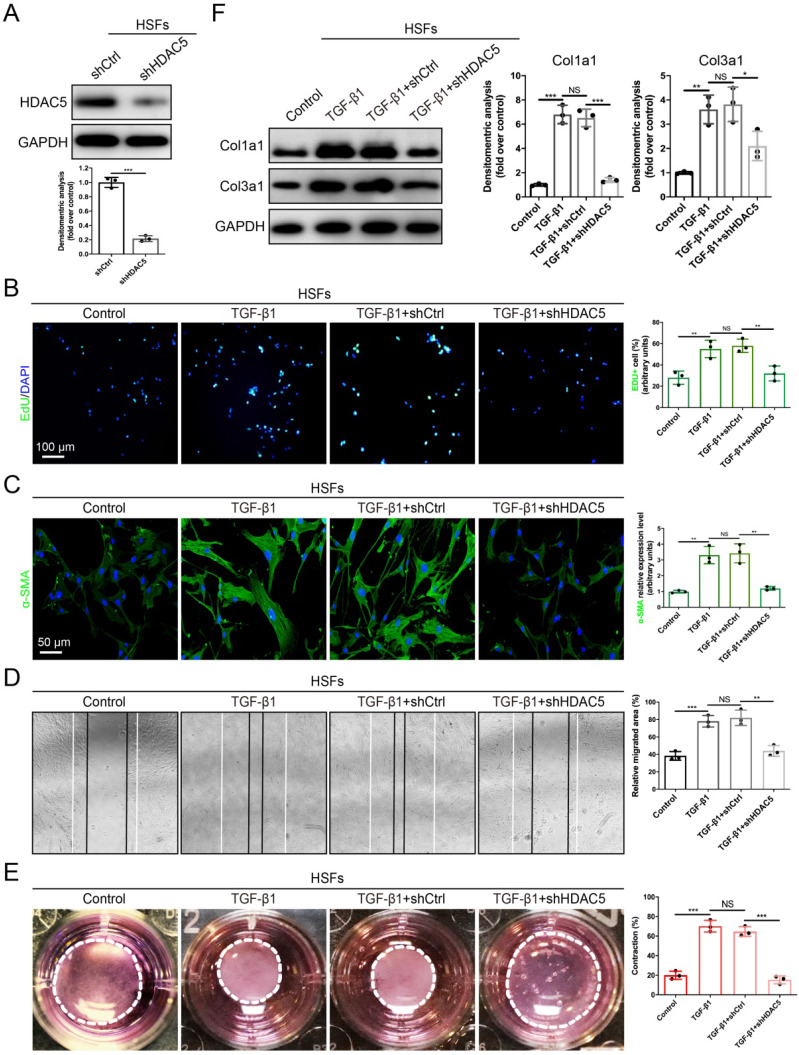
** HDAC5 knockdown inhibits TGF-β1-induced HSF activation. (A)** Identification of shHDAC5 efficiency in HSFs. **(B)** EdU (green) proliferation assay for cultured HSFs after incubation with TGF-β1 for 24 h. (Scale bar = 100 µm). **(C)** Images and quantification of immunofluorescence staining for α-SMA in different groups. α-SMA is labeled in green. (Scale bar = 50 µm). **(D)** Images and quantification of wound healing assays in different groups 12 h after the addition of TGF-β1. **(E)** Images and quantification of collagen gel contraction assays in different groups on Day 3 after TGF-β1 addition. Dashed lines indicate the areas of collagen gel. **(F)** The protein levels of collagen I and III in HSFs pretreated with TGF-β1 for 24 h. Data are presented as the means with SEs (n = 3 independent experiments). *P < 0.05, **P < 0.01, ***P < 0.001, NS = not significant.

Fibroblast activation plays a significant role in HS formation 29. Because we proved that HDAC5 was highly expressed in activated fibroblasts, we next evaluated the role of HDAC5 in the biological behaviors of fibroblasts. The EdU proliferation assay showed that the percentage of EdU-positive cells increased significantly after TGF-β1 incubation, while HDAC5 KD in human HS-derived fibroblasts (HSFs) (Fig. 3A) strongly attenuated TGF-β1-induced fibroblast proliferation (Fig. 3B). In addition, the immunofluorescence staining demonstrated that HDAC5 knockdown significantly inhibited α-SMA expression induced by TGF-β1, suggesting the important effect of HDAC5 on fibroblast activation (Fig. 3C). In addition, once HDAC5 was knockdown by shRNA, the enhancing effect of TGF-β1 on HSF migration, contraction and collagen secretion was obviously blocked (Fig. 3D-F). Consistent with the results of HSFs, embryonic fibroblasts (MEFs) from HDAC5 KO mice also displayed obvious resistance to TGF-β1-induced cell proliferation, activation, migration, contraction and collagen production (Supplementary Fig. S3A-E).

### HDAC5-mediated Smad7 silencing is critical for TGF-β1-induced fibroblast activation* in vitro*

**Figure 4:**
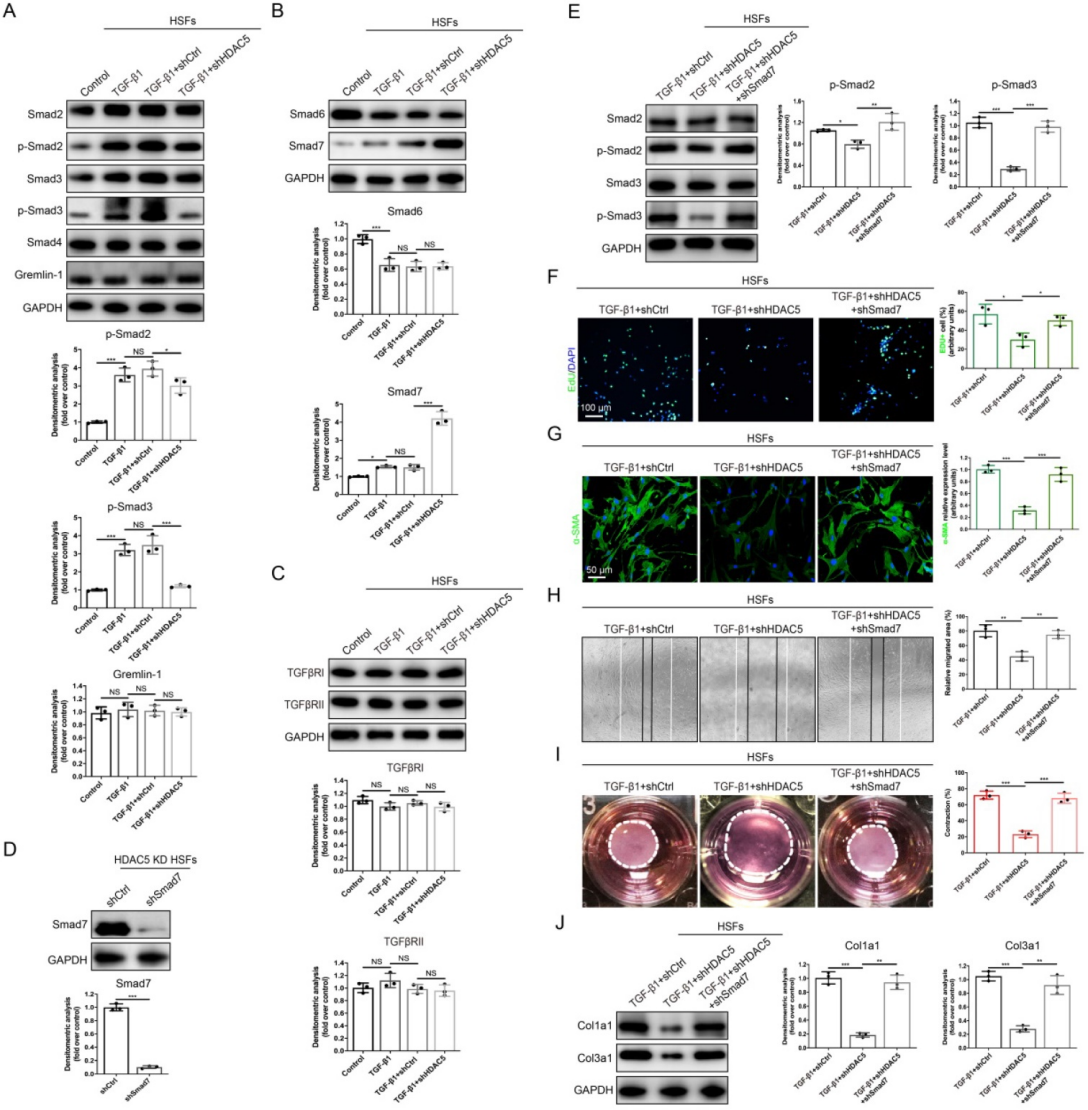
** HDAC5-mediated Smad7 silencing is critical for TGF-β1-induced HSF activation. (A-C)** Western blot assay of phosphorylated and total Smad2 and Smad3 and total Smad4, Smad6, Smad7, Gremlin 1, TGFβRI and TGFβRII in different groups. Samples were collected 12 h after the addition of TGF-β1. **(D)** Identification of shSmad7 efficiency in HSFs with HDAC5 KD. **(E)** The protein levels of phosphorylated and total Smad2 and Smad3 in MEFs pretreated with TGF-β1 for 12 h. **(F)** EdU (green) proliferation assay of cultured HSFs after incubation with TGF-β1 for 24 h (scale bar = 100 µm). **(G)** Images and quantification of immunofluorescence staining for α-SMA in different groups. α-SMA is labeled in green. (Scale bar = 50 µm). **(H)** Images and quantification of wound healing assays in different groups 12 h after TGF-β1 addition. **(I)** Images and quantification of collagen gel contraction assays in different groups on Day 3 after TGF-β1 addition. Dashed lines indicate the areas of collagen gel. **(J)** The protein levels of collagen I and III in HSFs pretreated with TGF-β1 for 24 h. Data are presented as the means with SEs (n = 3 independent experiments). *P < 0.05, **P < 0.01, ***P < 0.001, NS = not significant.

To further explore the specific mechanisms underlying the regulation of TGF-β1-induced fibroblast activation by HDAC5, we next evaluated whether HDAC5 is required for the activation of TGF-β/Smads signaling. Western blot analysis demonstrated that upon HDAC5 KD/KO, TGF-β1-induced phosphorylation of Smad2 and Smad3 was obviously blocked without having a significant impact on total Smad2/3 and Smad4 expression (Fig. 4A and Supplementary Fig. S4A). Meanwhile, a recently discovered profibrotic protein in skin fibrosis, Gremlin 1 30, was also detected. Our data demonstrated that TGF-β1 did not increase the protein level of Gremlin 1 (Fig. 4A and Supplementary Fig. S4A) which was consistent with a previous study 31. Also, HDAC5 silencing had no significant effect on the expression of Gremlin 1 (Fig. 4A and Supplementary Fig. S4A). Additionally, HDAC5 KD/KO significantly upregulated Smad7 expression and did not affect Smad6 expression (Fig. 4B and Supplementary Fig. S4B). In addition, the expression of TGFβRI/II was not notably altered by HDAC5 KD/KO (Fig. 4C and Supplementary Fig. S4C).

Since Smad7 has been shown to antagonize the signaling mediated by Smad2/3, we hypothesized that the promotion of Smad2/3 phosphorylation may be associated with the inhibition of Smad7 by HDAC5. Consequently, Smad7 was knock down by shRNA in HDAC5 KD HSFs (Fig. 4D) and HDAC5 KO MEFs (Supplementary Fig. S4D). Western blot analysis showed that Smad7 knockdown remarkably rescued the HDAC5 KD/KO-mediated down-regulation of p-Smad2 and p-Smad3 (Fig. 4E and Supplementary Fig. S4E). In addition, the negative effect of HDAC5 KD/KO on fibroblast proliferation, activation, migration, contraction and collagen production was rescued after Smad7 knockdown (Fig. 4F-J and Supplementary Fig. S4F-J). These results suggested that HDAC5-mediated Smad7 silencing played an important role in TGF-β1-induced fibroblast activation.

### HDAC5 promotes HS formation through Smad7 repression *in vivo*

**Figure 5:**
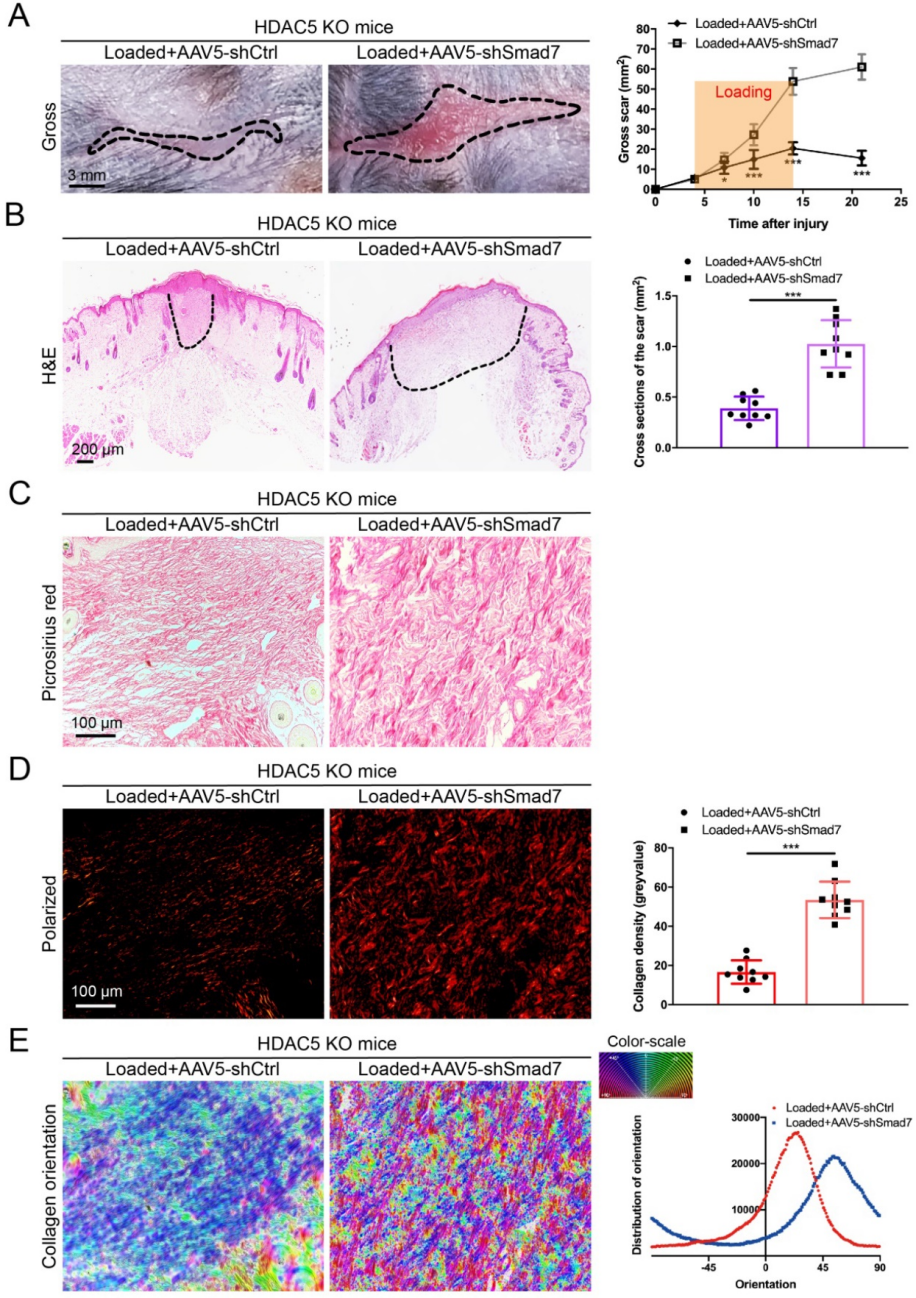
** Smad7 KD promotes hypertrophic scar formation in HDAC5 KO mice. (A)** Images of scars 14 days post-incision and gross area quantification at all examined time points. (Scale bar = 3 mm). **(B)** Images of H&E-stained sections and cross-section size quantification in different groups. The dashed lines outline the scar (scale bar = 200 µm). **(C, D)** Images of picrosirius red-stained sections under ordinary light and polarized light and collagen density quantification in different groups (scale bar = 100 µm). **(E)** The orientation of collagen fibers was quantified from picrosirius red using Orientation J software. The color representation reflects the different orientations. Data are presented as the mean ± SD (n = 9 biologically independent animals). *P < 0.05, ***P < 0.001.

To further confirm that HDAC5-mediated Smad7 silencing is critical for HS formation, Smad7 was knockdown in HDAC5 KO mice through the AAV system (Supplementary Fig. S5A). After AAV5-shSmad7 was administered, mice exhibited a significantly increased average scar area at each time point compared with AAV5-shCtrl-treated mice (Fig. 5A). Similarly, the cross-sectional size and collagen density of the scar were dramatically increased in AAV5-shSmad7-treated HDAC5 KO mice (Fig. 5B-E). These results indicated that Smad7 KD significantly promoted HS formation in HDAC5 KO mice, implying that HDAC5 regulated HS formation through Smad7 repression *in vivo.*

### HDAC5 diminishes the transcriptional activity of MEF2A on the Smad7 promoter

**Figure 6:**
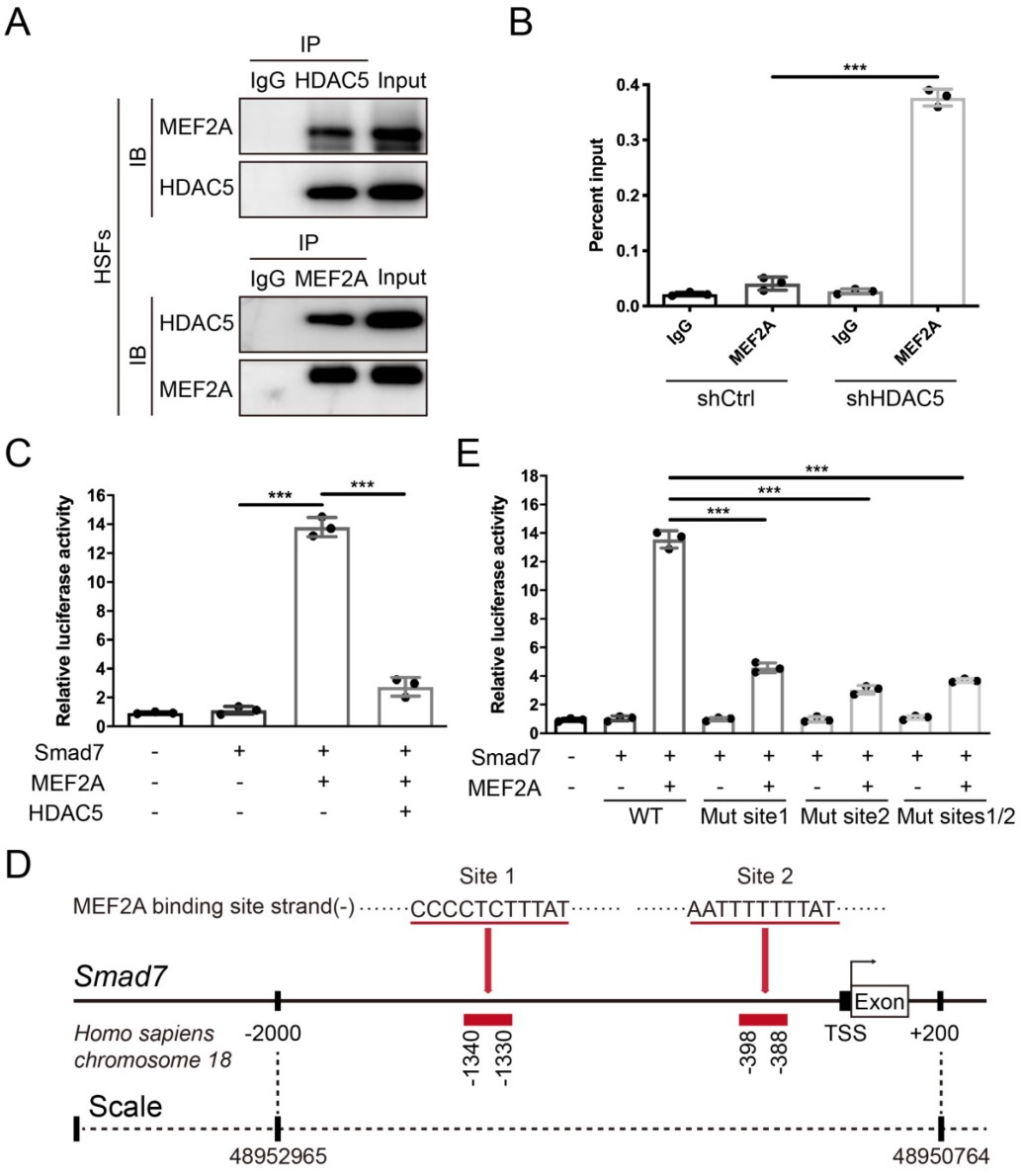
**HDAC5 interacts with MEF2A and diminishes its transcriptional activity on the Smad7 promoter region. (A)** Co-IP assay between HDAC5 and MEF2A in HSFs. **(B)** ChIP assay confirmation of the binding of MEF2A to the Smad7 promoter region in the shCtrl and shHDAC5 groups of HSFs. DNA immunoprecipitated by MEF2A antibody or immunoglobulin G (IgG CTL) was amplified by RT-qPCR using primers for the Smad7 promoter. **(C)** Activation of the Smad7 promoter luciferase reporter by MEF2 and attenuation by HDAC5 in HSFs. **(D)** Prediction of MEF2A-binding sites in the Smad7 promoter region using JASPAR software. **(E)** Effects of MEF2A-binding site mutations in the Smad7 promoter on transcriptional activation by MEF2A. Data are presented as the means with SEs (n = 3 independent experiments). ***P < 0.001.

After confirming that HDAC5 promoted HS formation by inhibiting Smad7 expression, we next examined its underlying mechanism. Previous studies have found that the interaction between the transcription factor MEF2 and HDAC5 can induce gene silencing 32. In vertebrates, the MEF2 family consists of MEF2A, B, C and D, and MEF2A is highly expressed in human skin tissue according to the Human Protein Atlas database (https://www.proteinatlas.org). Thus, we conducted a Co-IP assay and revealed an interaction between HDAC5 and MEF2A in both HSFs and MEFs (Fig. 6A and Supplementary Fig. S5B). In addition, with the ChIP certification followed by qPCR (ChIP-qPCR), we confirmed that HDAC5 KD induced the binding of MEF2A to the Smad7 promoter region (Fig. 6B and Supplementary Fig. S5C). The luciferase reporter assay also demonstrated that MEF2A activated the transcription of Smad7, and HDAC5 overexpression significantly inhibited the activating effect of MEF2A on Smad7 transcription (Fig. 6C and Supplementary Fig. S5D). Using JASPAR software, we predicted two MEF2A binding sites in the Smad7 promoter region (*Homo sapiens*, -1340-1330 and -398-388) (Fig. 6D). Further binding site mutation experiments showed that both individual and combined mutations impaired the binding of MEF2A to the Smad7 promoter region, suggesting that MEF2A may target these two sites (Fig. 6E). As NF-κB has also been reported could interact with HDAC5 33-35, we detected their interaction in both HSFs and MEFs. Results showed that there is no interaction between HDAC5 and NF-κB in these two cells (Supplementary Fig. S6A, B). Also, the ChIP-qPCR assay demonstrated that HDAC5 KD did not induce the binding of NF-κB to the Smad7 promoter region (Supplementary Figure S6C, D). In addition, studies have shown that the acetylation level of Smad7 is associated with its stability 36, and that HDAC5 can regulate protein stability through non-histone protein deacetylation 37. In this study, we did not detect an interaction between HDAC5 and Smad7, and HDAC5 KD/KO did not affect Smad7 acetylation in HSFs and MEFs before or after TGF-β1 addition (Supplementary Fig. S7).

### HDAC4/5 inhibitor LMK235 attenuates hypertrophic scar formation

**Figure 7:**
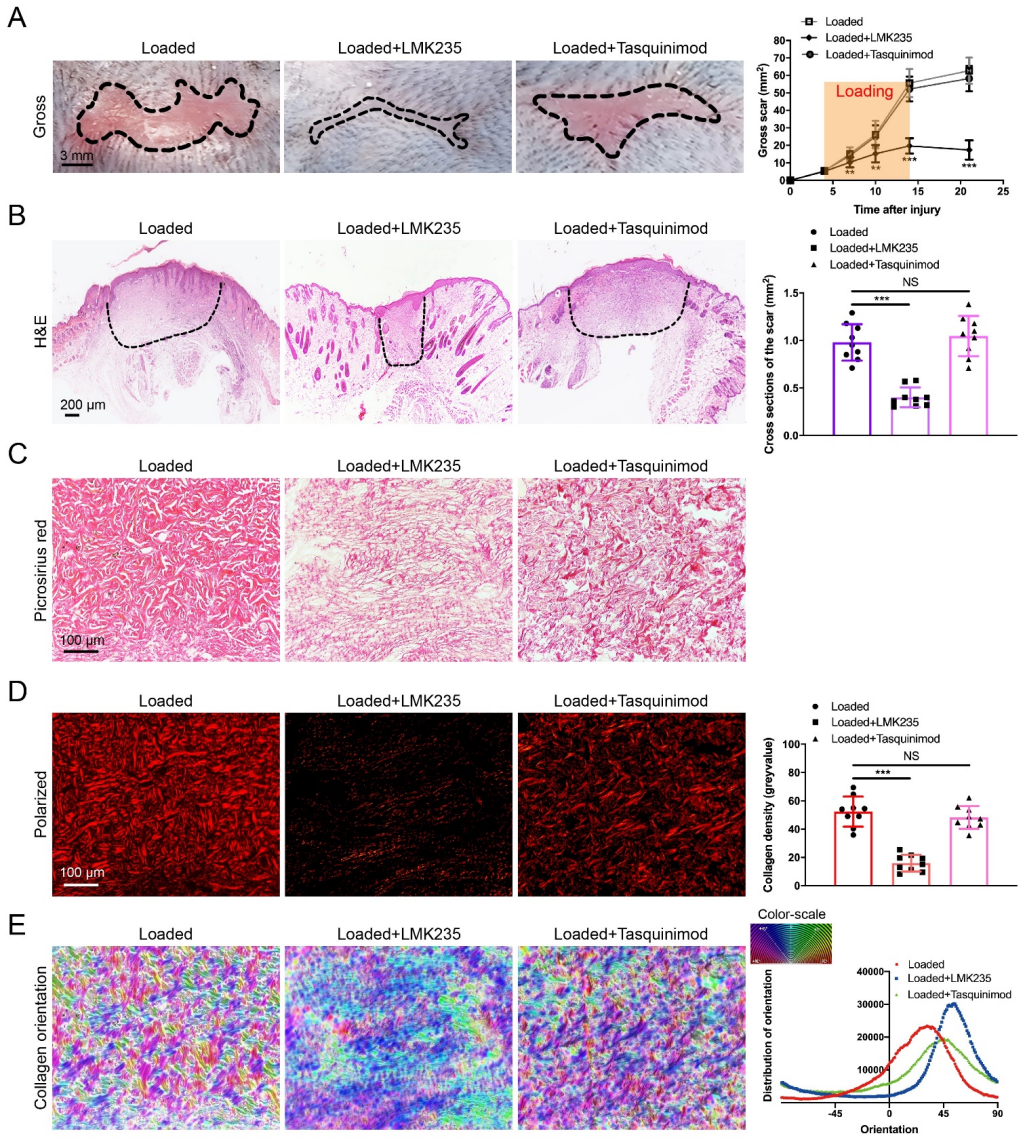
** LMK235 attenuates hypertrophic scar formation *in vivo*. (A)** Images of scars 14 days postincision and gross area quantification at all examined time points (scale bar = 3 mm). **(B)** Images of H&E-stained sections and cross-section size quantification in different groups. The dashed lines outline the scar (scale bar = 200 µm). **(C, D)** Images of picrosirius red-stained sections under ordinary light and polarized light and collagen density quantification in different groups (scale bar = 100 µm). **(E)** The orientation of collagen fibers was quantified from picrosirius red using Orientation J software. The color representation reflects the different orientations. Data are presented as the mean ± SD (n = 9 biologically independent animals). **P < 0.01, ***P < 0.001, NS = not significant.

As HDAC5 plays a crucial role in promoting HS formation, we examined the effects of HDAC inhibitors in a mouse hypertrophic scar model. Since there is currently no inhibitor specifically targeting HDAC5, the HDAC4/5 (LMK235) co-inhibitor and the HDAC4 specific inhibitor (Tasquinimod) were used for evaluation. As shown in Fig. 7A-E, the gross scar area, scar cross-sectional size and collagen density were significantly decreased and the disorder in collagen fibril orientation was substantially reduced in the LMK235-injected group compared with the control group. In contrast, the HDAC4 inhibitor Tasquinimod had no obvious effect on HS formation (Fig. 7A-E). These results further confirmed that HDAC5 participated in HS formation and could be a promising target for HS treatment.

## Discussion

In the present study, we identified the pathological role of HDAC5 in HS formation and discovered its role as a new regulator of the canonical TGF-β/Smads pathway. During HS formation, HDAC5 interacts with the transcriptional factor MEF2A, blocking its binding to the Smad7 promoter region. This causes the down-regulation of Smad7 expression, leading to an imbalance in negative feedback regulation of the TGF-β/Smads pathway, thereby enhancing fibroblast activation in multiple aspects and finally augmenting HS formation.

Current studies have shown that HDACs are closely associated with fibroproliferative disease 38. HDAC3 aberration can inhibit Klotho transcription and promote renal fibrosis 39. The protein levels of HDAC8 were observed to be increased in mice with bile duct ligation and patients with cholestatic liver injury and fibrosis 40. However, few studies have focused on the role of HDAC5 in skin fibrosis. In this study, we found that HDAC5 is overexpressed both in human and mouse HS tissues and that HDAC5 KO significantly attenuates HS formation in a mouse model. In line with our study, researchers have shown that HDAC5 KD followed by ATAC-seq in scleroderma led to the identification of key HDAC5-regulated genes involved in fibrosis 22. Furthermore, HDAC5 KD/KO remarkably ameliorated phenotypic changes associated with fibroblast activation, such as α-SMA up-regulation, ECM production, proliferation increase and migration and contraction enhancement. Consistent with our study, HDAC5 was also found to promote the proliferation and migration of various cells, such as tumor cells 37, smooth muscle cells 41 and epithelial cells 42.

Substantial evidence has suggested that TGF-β/Smads signaling plays a key role in the progression of tissue fibrosis 43. Smad7 is well known to be an important feedback inhibitor of TGF-β signaling, and its role in skin fibrosis has been revealed 28. Here, we demonstrated that HDAC5 interferes with the inhibitory effects of Smad7 on the TGF-β pathway, yet it does not affect Smad6. Similarly, Pusoon Chun showed that another class IIa HDAC, HDAC4, can increase the expression of multiple profibrotic molecules by inhibiting Smad7 in renal fibrosis 44. In addition, our results confirmed that HDAC5-induced Smad7 inhibition enhances the Smads signaling by increasing Smad2/3 phosphorylation, but not by regulating the Co-Smad, Smad4, or TGF-β receptors. In contrast to our results, a previous study showed that in diabetic nephropathy, overexpression of HDAC5 significantly attenuated TGF-β1-induced PAI-1 and p21 expression, which indicated the negative regulatory role of HDAC5 in TGF-β1 signaling 45. The discrepancy between the two studies may be due to HDAC5 playing different roles in regulating the TGF-β pathway in different diseases. Therefore, the network that exists between TGF-β signaling and HDAC5 is worthy of future investigation.

Next, we primarily explored the specific mechanism by which HDAC5 regulates Smad7 protein level. Generally, HDAC5 regulates gene expression in two ways: 1) by binding to transcription factors, such as MEF2, CtBP and HP1 46, through its N-terminal adapter domain and regulating their transcriptional activity, or 2) by targeting the SMRT/NCoR-HDAC3 complex to a specific subcellular location through its C-terminal deacetylase domain 47. In addition, HDAC5 can also affect protein stability through deacetylation of non-histone proteins, such as transcription factors and cytoplasmic proteins 37. In the present study, we revealed that HDAC5 inhibits the transcription of Smad7 through its interaction with MEF2A. The MEF2 proteins belong to the MADS (MCM-1-agamous-deficiens-serum response factor) family of transcriptional regulators 48. Previous studies have demonstrated that the interaction between MEF2 and HDAC5 plays a vital role in multiple physiological and pathological processes. It has been shown that HDAC5 blocks myogenesis by associating with and inhibiting the activity of the MEF2 transcription factor 49. Another study demonstrated that fluid shear stress stimulates nuclear export of HDAC5 in endothelial cells, releasing its repression of MEF2 and thus modulating the flow anti-inflammatory effect in endothelial cells 50. Our Co-IP assay and luciferase reporter assay revealed an interaction between HDAC5 and MEF2A in both HSFs and MEFs, and HDAC5 inhibited the activating effect of MEF2A on Smad7 transcription. Moreover, we discovered two MEF2A binding sites of in the Smad7 promoter region (-1340-1330 and -398-388). Consistent with our findings, one study has shown that a small molecule (AM-001) can inhibit cardiac hypertrophy and fibrosis by interfering with the binding of HDAC5 and MEF2 51.

In general, we revealed the crucial role of HDAC5 in the progression of hypertrophic scar formation. More essentially, we demonstrated a detailed mechanism underlying fibroblast activation and skin fibrosis through the interaction between HDAC5 and MEF2, in which HDAC5 inhibited MEF2-mediated Smad7 transcription, thus inducing activation of the TGF-β/Smads signaling. Additionally, we confirmed that the HDAC4/5 inhibitor LMK235 significantly attenuates hypertrophic scar formation. HDAC5 could serve as a promising therapeutic target for hypertrophic scar and other fibroproliferative disorders.

## Supplementary Material

Supplementary figures and tables.

## Abbreviations

TGF-β: Transforming growth factor-β
HDAC: histone deacetylase
HS: hypertrophic scar
KO: knockout
KD: knockdown
MEF2A: myocyte enhancer factor 2A
ECM: extracellular matrix
EMT: epithelial-mesenchymal transition
HDACi: HDAC inhibitors
SSc: systemic sclerosis
HSFs: hypertrophic scar fibroblasts
MEFs: Mouse embryonic fibroblasts
sgRNAs: single guide RNAs
WT: wide-type
RT-qPCR: quantitative real-time PCR
H&E: hematoxylin and eosin
EdU: 5-Ethynyl-2′-deoxyuridine
AAV: adeno-associated virus
Co-IP: Co-immunoprecipitation
ChIP: Chromatin immunoprecipitation
