# Supplementary material for: HDAC5-mediated Smad7 silencing through MEF2A is critical for fibroblast activation and hypertrophic scar formation
Source: Int J Biol Sci. 2022 Sep 11;18(15):5724–39. doi: 10.7150/ijbs.76140 (PMC9576526; doi:10.7150/ijbs.76140)
Supplement: Supplementary file 1 — Supplementary figures and tables. [file ijbsv18p5724s1.pdf]

Supplementary Material

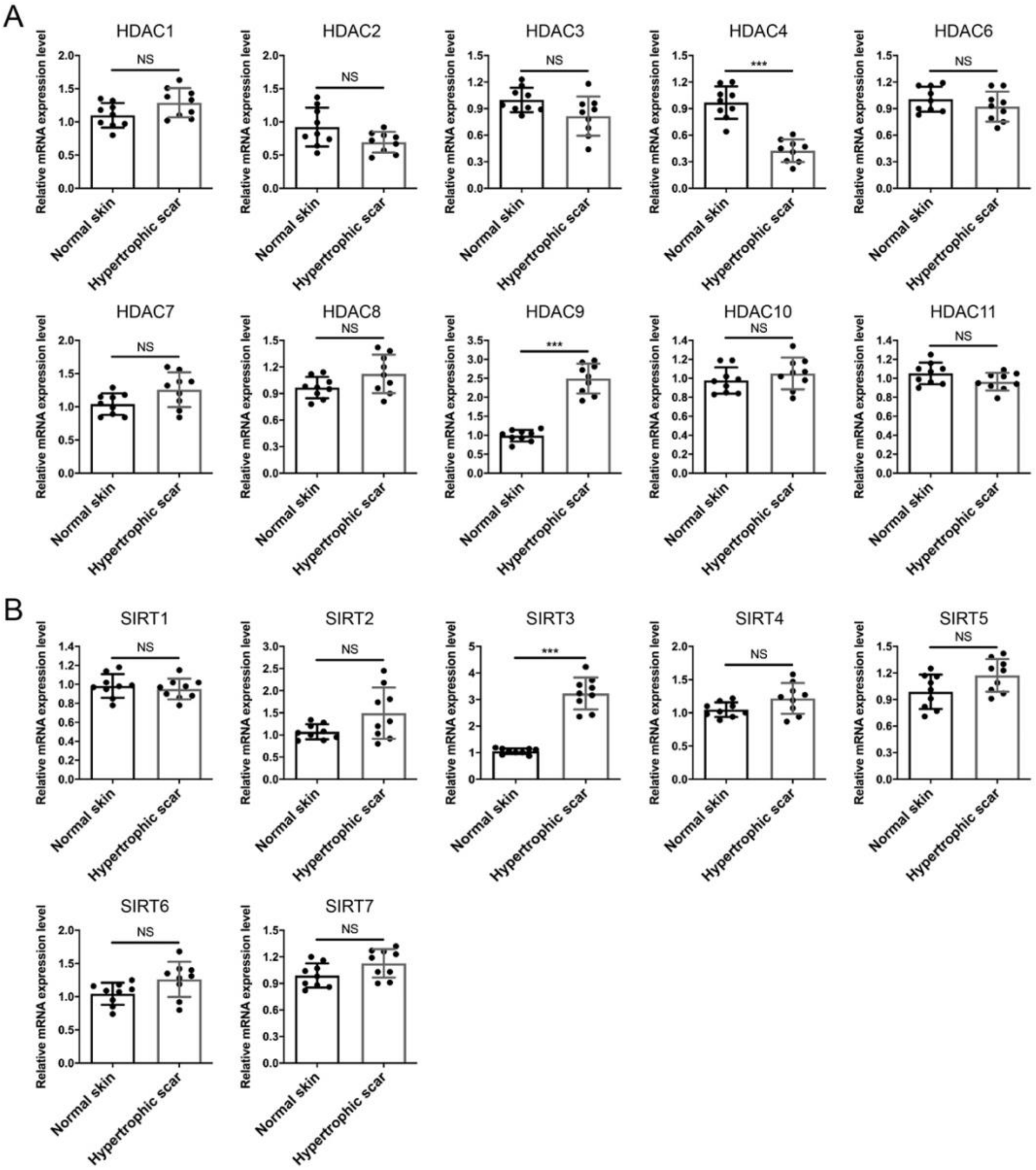

Supplementary Figure S1 The mRNA levels of HDAC family members in mice normal skin and HS tissues. (A) The mRNA levels of HDAC1-4 and HDAC6-11 in mice normal skin and HS tissues. (B) The mRNA levels of SIRT 1-7 in mice normal skin and HS tissues. Data are presented as the means with SEs (n = 3 independent experiments). ***$P < 0.001$, NS = not significant.

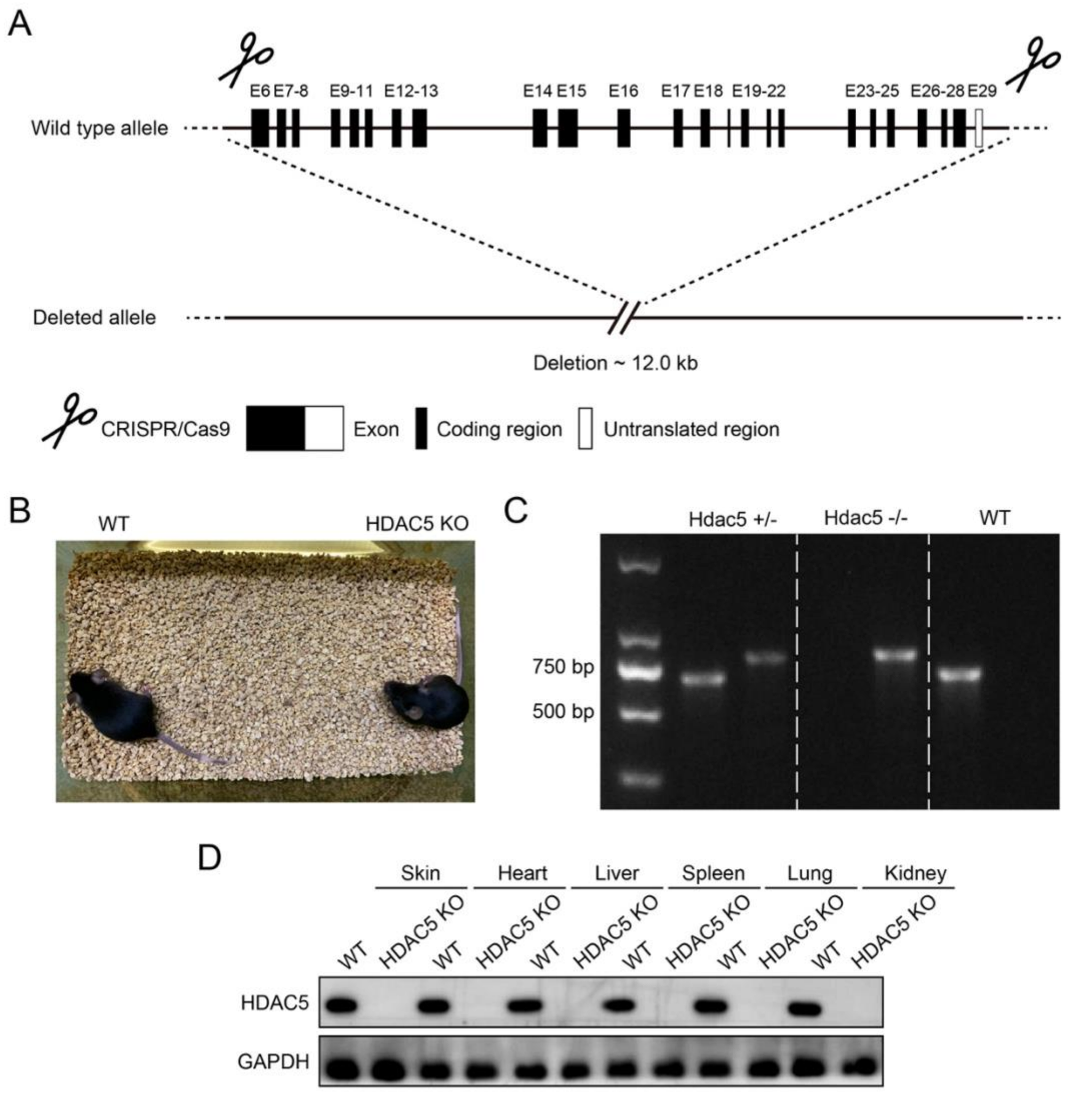

Supplementary Figure S2 Generation of HDAC5 KO mice. (A) Schematic illustration of the targeting strategy for generating HDAC5 KO mice. (B) Representative photographs of 8-week-old WT and HDAC5 KO mice. (C) Genotyping of heterozygous (*Hdac5+/-*), homozygous (*Hdac5-/-*), and WT mice. (D) HDAC5 protein levels in different tissues, including the skin, heart, liver, spleen, lung and kidney, from WT and HDAC5 KO mice.

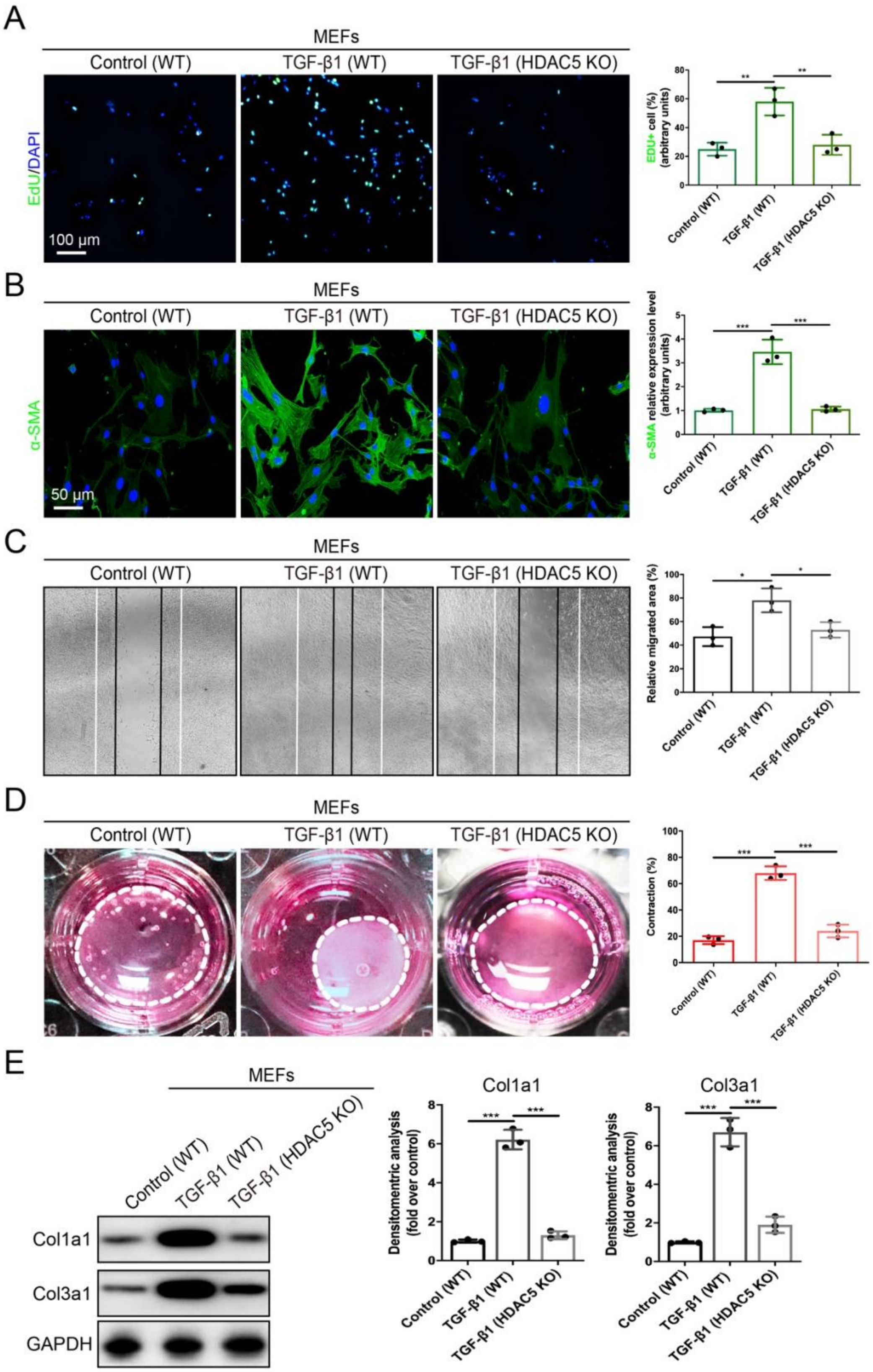

Supplementary Figure S3 HDAC5 KO inhibits TGF-β1-induced MEF activation.

(A) EdU (green) proliferation assay for cultured MEFs after incubation with TGF-β1

for 24 h. (Scale bar = 100 μm). (B) Images and quantification of immunofluorescence staining for α-SMA in different groups. α-SMA is labeled in green. (Scale bar = 50 μm). (C) Images and quantification of wound healing assays in different groups 12 h after TGF-β1 addition. (D) Images and quantification of collagen gel contraction assays in different groups on Day 3 after TGF-β1 addition. Dashed lines indicate the areas of collagen gel. (E) The protein levels of collagen I and III in MEFs pretreated with TGF-β1 for 24 h. Data are presented as the means with SEs (n = 3 independent experiments). *$P < 0.05$, **$P < 0.01$, ***$P < 0.001$.

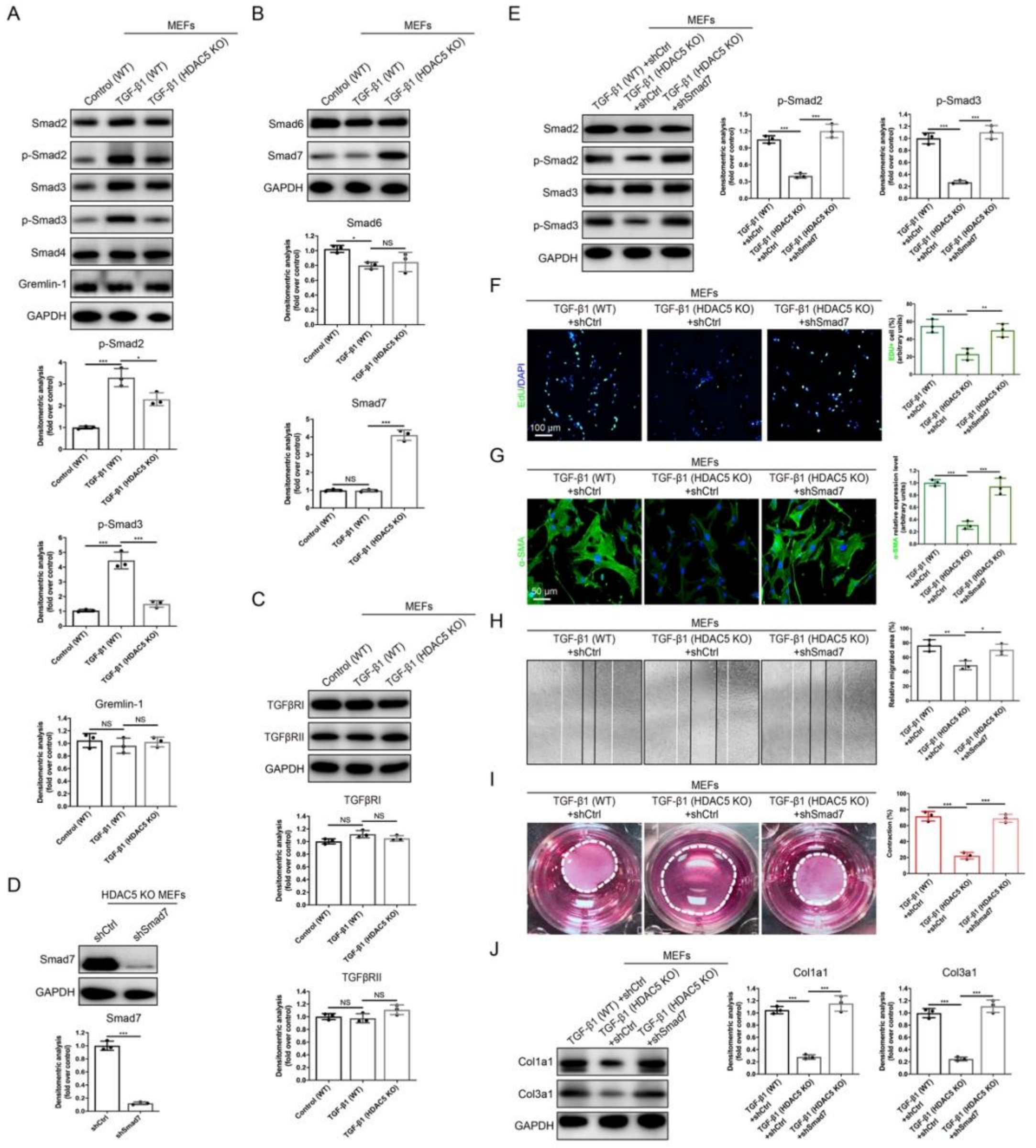

Supplementary Figure S4 HDAC5-mediated Smad7 silencing is critical for TGF-β1-induced MEF activation. (A-C) Western blot assay of phosphorylated and total Smad2 and Smad3 and total Smad4, Gremlin 1, Smad6, Smad7, TGFβRI and TGFβRII in different groups. Samples were collected 12 h after the addition of TGF-β1. (D) Identification of shSmad7 efficiency in HDAC5 KO MEFs. (E) The protein levels of phosphorylated and total Smad2 and Smad3 in MEFs pretreated with TGF-β1 for 12 h. (F) EdU (green) proliferation assay for cultured MEFs after incubation with TGF-β1

for 24 h. (Scale bar = 100 μm). (G) Images and quantification of immunofluorescence staining for α-SMA in different groups. α-SMA is labeled in green. (Scale bar = 50 μm). (H) Images and quantification of wound healing assays in different groups 12 h after TGF-β1 addition. (I) Images and quantification of collagen gel contraction assays in different groups on Day 3 after TGF-β1 addition. Dashed lines indicate the areas of collagen gel. Data are presented as the means with SEs (n = 3 independent experiments). *$P < 0.05$, **$P < 0.01$, ***$P < 0.001$, NS = not significant.

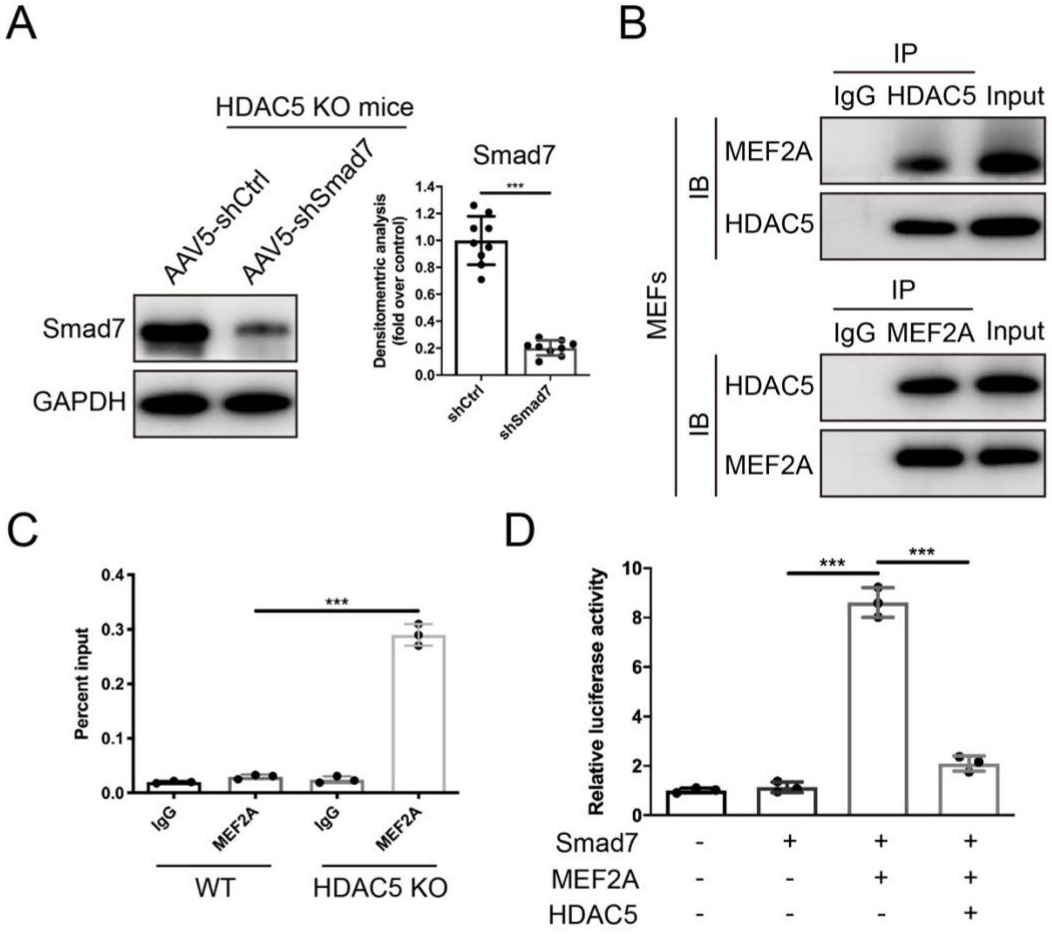

Supplementary Figure S5 HDAC5 interacts with MEF2A and diminishes its transcriptional activity on the Smad7 promoter region. (A) Validation of AAV5-mediated KD efficiency of Smad7 in HDAC5 KO mice using Western blot assay. (B) Co-IP assay between HDAC5 and MEF2A in MEFs. (C) ChIP assay confirmation of the binding of MEF2A to the Smad7 promoter region in WT MEFs and HDAC5 KO MEFs. DNA immunoprecipitated by MEF2A antibody or IgG was amplified by RT–qPCR using Smad7 primers. (D) Activation of the Smad7 promoter luciferase reporter by MEF2 and attenuation by HDAC5 in MEFs. Data are presented as the means with SEs (n = 3 independent experiments). ***$P < 0.001$.

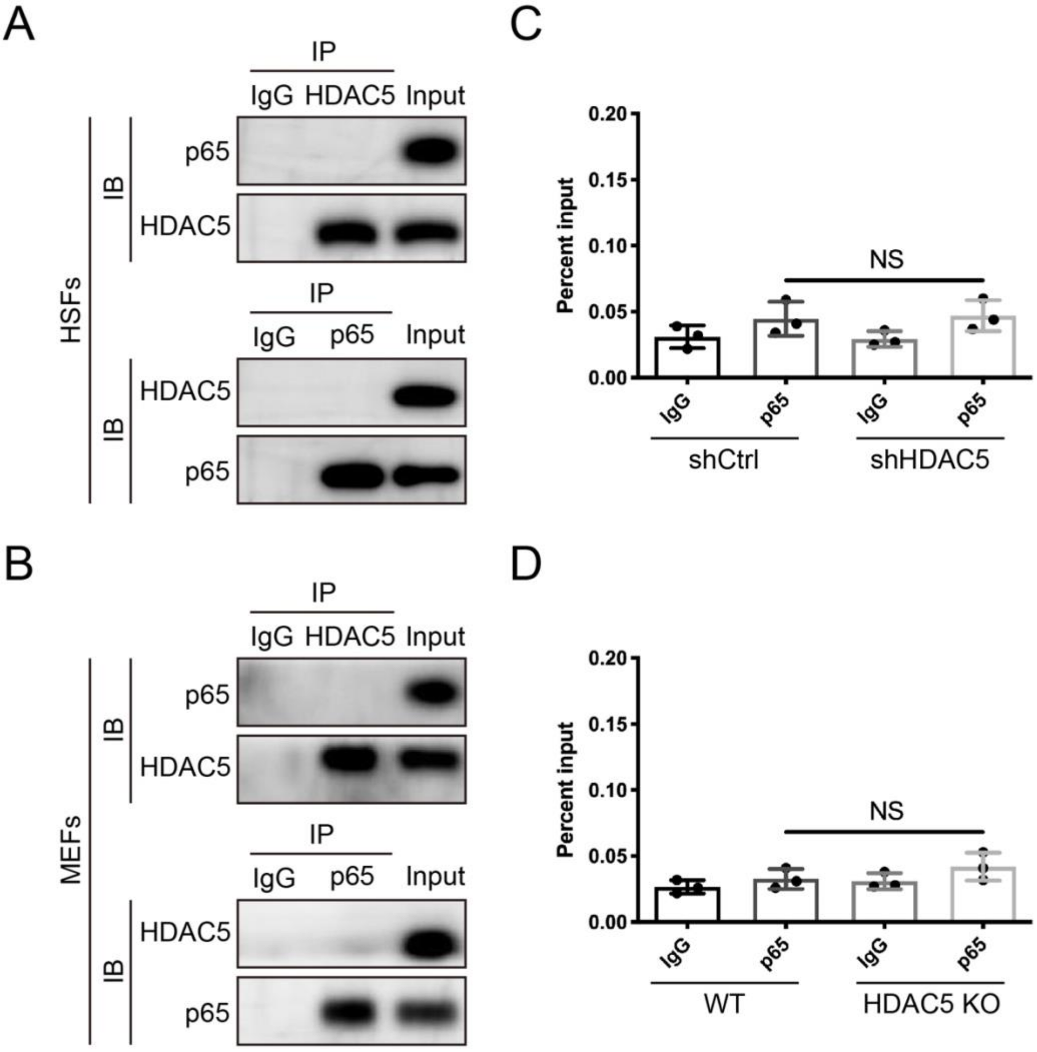

Supplementary Figure S6 HDAC5 has no interaction with NF-κB. (A, B) Co-IP assay between HDAC5 and NF-κB p65 in HSFs and MEFs. (C, D) ChIP assay test of the binding of NF-κB p65 to the Smad7 promoter region in HSFs and MEFs. DNA immunoprecipitated by NF-κB p65 antibody or IgG was amplified by RT–qPCR using Smad7 primers. Data are presented as the means with SEs (n = 3 independent experiments). NS = not significant.

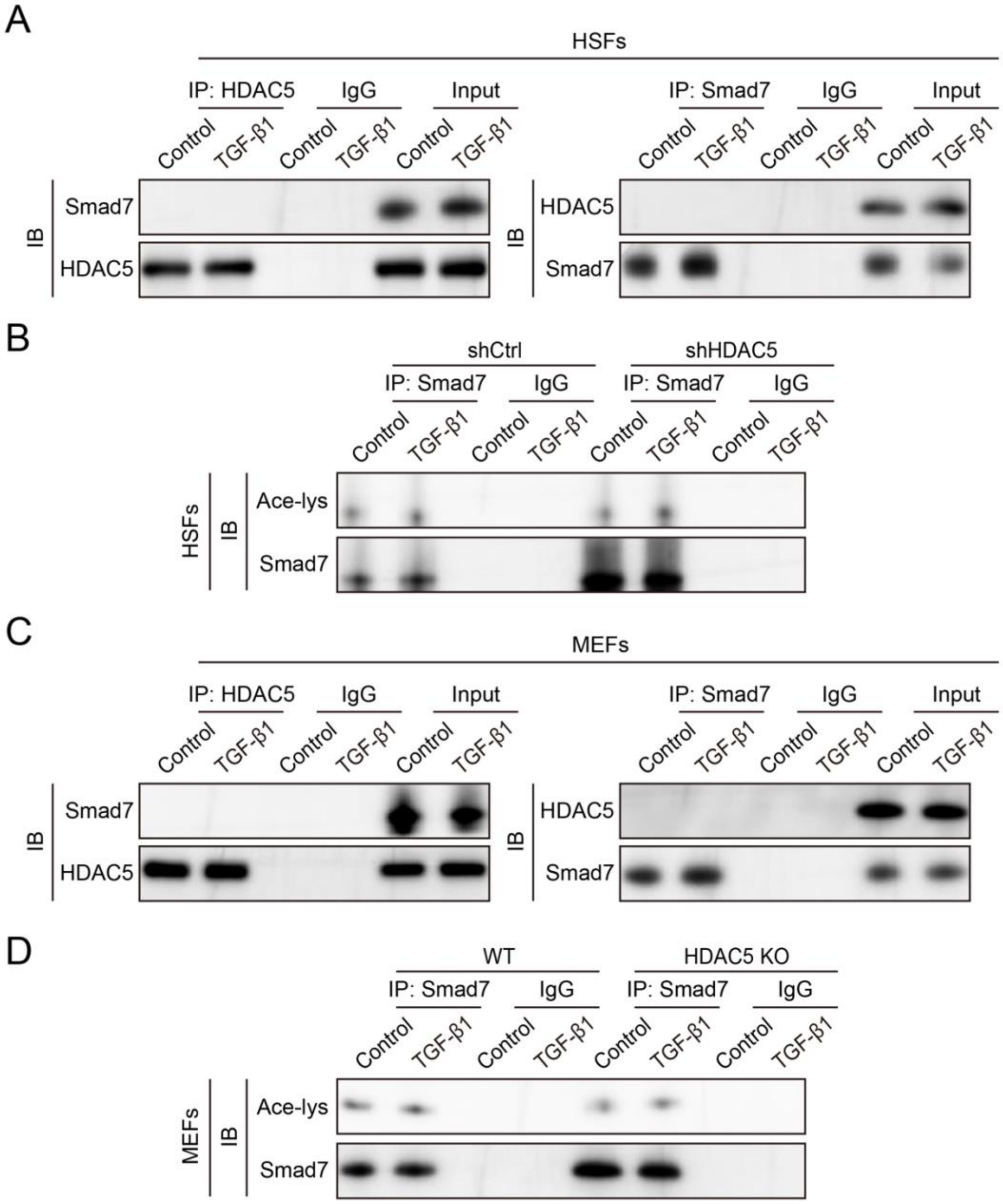

Supplementary Figure S7 HDAC5 does not affect Smad7 acetylation. (A) Co-IP assay between HDAC5 and Smad7 in HSFs with or without TGF-β1 treatment. (B) Smad7 acetylation levels in HSFs from different groups. HSFs were transfected with shCtrl/shHDAC5 and treated with or without TGF-β1. (C) Co-IP assay between HDAC5 and Smad7 in MEFs with or without TGF-β1 treatment. (D) Smad7 acetylation levels in WT and HDAC5 KO MEFs treated with or without TGF-β1. (n = 3 independent experiments).

Supplementary Table S1. Volunteers' information

Volunteer	Sex	Age (years)	Localization	Time after trauma or burn (months)
1	M	37	Chest	8
2	F	25	Shoulder	12
3	F	32	Chest	10
4	M	18	Forehead	6
5	M	24	Shoulder	10
6	F	40	Trunk	12
7	F	27	Back	7
8	F	22	Ear	10
9	M	25	Back	11
10	M	33	Chest	6
11	M	36	Cheek	7
12	F	24	Trunk	9
13	F	31	Nose	6
14	M	29	Shoulder	12
15	F	36	Cheek	10
16	M	42	Trunk	10
17	M	30	Back	11
18	F	28	Shoulder	9
19	M	19	Forehead	7
20	F	26	Chest	12

Supplementary Table S2. PCR primers used in this study

Primers	Forward 5′-3′	Reverse 5′-3′
Mus musculus Gapdh	AGGTCGGTGTGAACGGATTTG	TGTAGACCATGTAGTTGAGGTCA
Mus musculus Hdac1	AGTCTGTTACTACTACGACGGG	TGAGCAGCAAATTGTGAGTCAT
Mus musculus Hdac2	GGAGGAGGCTACACAATCCG	TCTGGAGTGTTCTGGTTTGTCA
Mus musculus Hdac3	GCCAAGACCGTGGCGTATT	GTCCAGCTCCATAGTGGAAGT
Mus musculus Hdac4	CTGCAAGTGGCCCCTACAG	CTGCTCATGTTGACGCTGGA
Mus musculus Hdac5	TGCAGCACGTTTTGCTCCT	GACAGCTCCCCAGTTTTGGT
Mus musculus Hdac6	TCCACCGGCCAAGATTCTTC	CAGCACACTTCTTTCCACCAC
Mus musculus Hdac7	GGCAGGCTTACACCAGCAA	TGGGCAGGCTGTAGGGAATA
Mus musculus Hdac8	ACTATTGCCGGAGATCCAATGT	CCTCCTAAAATCAGAGTTGCCAG
Mus musculus Hdac9	GCGGTCCAGGTTAAAACAGAA	GCCACCTCAAACACTCGCTT
Mus musculus Hdac10	CCAGGGCATCCAGTATATCTTCA	CAACTCAGGATCAAACTCGAAGG
Mus musculus Hdac11	GTGTACTCACCACGTTACAACA	GCTCGTTGAGATAGCGCCTC
Mus musculus Sirt1	GCTGACGACTTCGACGACG	TCGGTCAACAGGAGGTTGTCT
Mus musculus Sirt2	GCCTGGGTTCCCAAAAGGAG	GAGCGGAAGTCAGGGATACC
Mus musculus Sirt3	ATCCCGGACTTCAGATCCCC	CAACATGAAAAAGGGCTTGGG
Mus musculus Sirt4	GTGGAAGAATAAGAATGAGCGGA	GGCACAAATAACCCCGAGG
Mus musculus Sirt5	CTCCGGGCCGATTCATTTCC	GCGTTCGCAAAACACTTCCG
Mus musculus Sirt6	ATGTCGGTGAATTATGCAGCA	GCTGGAGGACTGCCACATTA
Mus musculus Sirt7	CAGGTGTCACGCATCCTGAG	GCCCGTGTAGACAACCAAGT
Homo sapiens GAPDH	GGAGCGAGATCCCTCCAAAAT	GGCTGTTGTCATACTTCTCATGG

Homo sapien HDAC5	GGTGTGGTCTACGACACGTTC	GATCCGCTCGCACTTGCTAA
